# Signalling strategies and opportunistic behaviour: Insights from dark-net markets

**DOI:** 10.1371/journal.pone.0319794

**Published:** 2025-03-18

**Authors:** Filippo Andrei, Giuseppe Alessandro Veltri

**Affiliations:** 1 Department of Sociology and Social Research, University of Trento, Trento, Italy; 2 National University of Singapore and the Center for Behavioural and Implementation Science, Singapore, Singapore; University Putra Malaysia: Universiti Putra Malaysia, MALAYSIA

## Abstract

The emergence of social order on darknet markets presents social scientists with a unique puzzle. Because these markets operate outside of conventional regulatory frameworks, there is a lack of legitimate oversight to monitor transactions and protect users from opportunistic behaviour. While existing literature often examines the role of reputation in increasing sales, little attention has been paid to mechanisms that mitigate fraud. This study fills this gap by examining one of the largest known darknet platforms, Alphabay, which was operational from December 2014 to July 2017. Using two Generalised Additive Models (GAMs), results show that costly signals, such as a positive reputation, sellers’ seniority and escrow services, are inversely associated with fraudulent activity on darknet markets. Conversely, cheap signals, such as long product descriptions characterised by complex vocabulary and a positive tone, correlate positively with opportunistic behaviour. The study provides empirical support for signalling theory, by showing that costly signals are more difficult to fake or manipulate and can reduce fraud. Conversely, the study also demonstrates empirically that cheap signals, while potentially effective in initially generating trust among buyers, are associated with an increase in fraud and opportunistic behaviour.

## Introduction

The emergence of social order in the online marketplace is a challenge for social scientists because it involves the complicated orchestration of harmonised expectations between participants in the ever-changing landscape of the digital marketplace. The harmonisation of expectations between the parties involved in the transactions proves to be a crucial factor in the exchange’s success. In the context of the dynamics of the online marketplace, buyers struggle with the complexity and inherent uncertainty associated with assigning value to goods or services. This involves complicated social processes in setting standards to distinguish qualities [1–3] as buyers cannot immediately verify the quality of products. In addition, transactions become uncertain when actors cannot be sure that contracts or voluntary agreements will be honored [4]. In this context, the problem of cooperation and opportunistic behaviour arises from the social risks and uncertainties inherent in exchange, as actors have incomplete knowledge about the intentions of their exchange partners and product quality [2,5]. For these reasons, opportunistic behaviour and trust become a significant problem in computer-mediated transactions [6]. Therefore, sellers need to prove their reliability by demonstrating observable characteristics such as trustworthiness. Despite these challenges, the popularity of electronic peer-to-peer marketplaces has grown rapidly [7]. In the last decade, online marketplaces have even embraced illegal products thanks to technological tools such as Tor browsers and Bitcoin, guaranteeing anonymity. The problem of the social order of the market is even more pronounced in illegal online markets (also known as darknet markets or/crypto markets), especially because these markets operate outside the law and, therefore, have no authority to set uniform product quality standards and prevent fraud. Additionally, an illegal market is populated by individuals actively involved in unlawful activities and prone to transgressions, making them more inclined to break rules and commit fraud than traders on legal markets [8]. Last but not least, darknet markets, like most digital e-commerce platforms, face the problem of asymmetric information, as buyers cannot assess the quality of a product before purchasing it. Therefore, the issue of trust and opportunistic behaviour arises in this context, as actors can benefit from the transaction if they are willing to accept the risk associated with the conditions of darknet transactions [9]. Nevertheless, illicit online markets have proliferated through various channels, including the surface web (all websites accessible through a standard browser), the darknet (an encrypted network accessible only through anonymous browsers) and encrypted messaging applications installed on smartphones [10]. These markets enable trade in various goods and services such as weapons, stolen cards, fake documents, chemicals and counterfeit items; however, supply and demand are primarily focused on drugs. The most widespread type of illicit online market is a crypto market, which relies mainly on an audience of end users in Europe, North America and Oceania [11]. Cryptomarkets are located on the dark web and are accessible via cryptographic software that allows buyers and sellers to hide their identities from law enforcement [10,12]. This is achieved through the TOR network, which anonymises the internet traffic of dark web users [10]. In addition, traders use virtual currencies that allow them to conduct online transactions with a high degree of anonymity (an example of an offer on AlphaBay Market can be observed in S1 Fig). The organisational structure of the darknet market resembles what economists call a”platform economy” [13]. These websites provide a platform that is accessible to both buyers and sellers and, in return, charge a commission for brokering transactions and organising information and items. Platform management offers advantages for both parties: sellers can reach a wide audience of buyers, while buyers can compare different items before making a purchase [14]. Despite its illegal nature, the darknet market often has a high percentage of positive reviews, with most negative reviews being due to delivery problems [15,16]. What factors help to reduce opportunistic behaviour in this context? The existing literature on the darknet market focuses primarily on how reputation, status and escrow, together with repeated interactions, contribute to increasing sales by facilitating transactions between buyers and sellers [17–20]. However, as far as we are aware, there is no study that analyses the main factors contributing to the decrease or increase of fraud on the darknet market. In this paper, we fill this gap by applying signal theory and analysing the signals that decrease fraud in the Alphabay market, one of the largest darknet markets that has ever existed.

## Signalling theory and opportunistic behaviour

Signal theory, which has its roots in information economics and takes up concepts from biological signal theory, is applied in various areas digital realm, Internet recruitment website, online market, online game, and illegal activities [8,21–24]. Its main purpose is to clarify how the challenges arising from asymmetric information can be effectively addressed, especially in situations characterised by high uncertainty. The theory assumes that deliberate signaling is used to reveal otherwise unobserved characteristics to overcome information asymmetry and trust problems [25]. In signalling theory, "cheap” and "costly” signals refer to different types of cues that individuals use to convey information about their characteristics, intentions or trustworthiness [26]. Cheap signals, which are comparatively easy or inexpensive to produce or falsify, usually do not require a great deal of resources or effort. They may convey some information to the recipient, but their reliability in terms of the sender’s true characteristics or intentions is often limited. Conversely, costly signals require a significant investment of resources, effort or risk on the part of the sender. Due to their high cost, they serve as reliable indicators of the sender’s true characteristics or intentions by demonstrating commitment or capability and increasing credibility with the recipient. Cheap signals (or cheap talk [27]) include verbal assurances, simple gestures or superficial statements, while costly signals include investment in education or training, significant financial investment or physically demanding tasks. Signalling theory has been used to study the trade in illegal goods and services on various platforms, including illegal card forums and darknet markets. For example, Décary-Hétu et al. discovered [28] a positive correlation between longevity and criminal opportunity, suggesting a link between the longevity of criminal actors in online carding forums and criminal reliability. While others [29] emphasized the importance of long-term engagement in online card market forums as a signal of trust, previous studies have also identified several signals of trust within these illicit markets. These signals include not only the duration of sellers’ activity on the sites, but also their status or rank and various indicators of criminal performance [30]. These findings contribute to a comprehensive understanding of how signalling theory works in the context of illegal activity and sheds light on the intricate dynamics of trust and reliability in these unconventional marketplaces. In the context of market transactions on the darknet, trust can be conceptualised as a three-part relationship [31] expressed as follows: ‘A trusts B with respect to X’ [32,33]. Here, A is the trustor who decides whether to trust; B is the trustee, the recipient of the trust, who decides whether to honour it; and X represents the content of the trust relationship [34]. The trustor, who must decide whether to trust the trustee concerning X, faces a strategic dilemma. If both parties cooperate, both benefits, while mutual non-trust leads to zero gain. However, if the settlor places trust and the trustee fail to honour it, the settlor suffers a loss, and the trustee gains disproportionately. Four basic elements characterise this trust relationship [31,35]. Firstly, the settlor’s vulnerability arises when relinquishing control over certain resources to the trustee [36]. This vulnerability is characterised by the fact that the interaction involves a stake in which both parties stand to lose or gain something, be it tangible or intangible. Furthermore, the delayed observation of trust outcomes exacerbates the vulnerability of the trust relationship [37]. A time gap appears between the trustor expressing trust and the trustee deciding to honour it, leading to uncertainty for the trustor. This delay is reflected in darknet platforms, where buyers must trust sellers before they know whether their trust will be honoured. Drug transactions across platforms including Empty Cell, Silk Road, Silk Road 2.0 and Evolution and found that only 1.2–2.9 percent of reviews were negative [38]. Additionally, 1.8–3.7 percent were neutral, indicating that 94.5–96.9 percent of transactions were positively rated. Similarly, results were presented by [16], showing that the recension in Silk Road, Alphabay, and Hansa was 97 percent, 96 percent, and 97 percent, respectively, positive. This means that sellers rarely abuse the trust buyers place in them. Considering that darknet market platforms cannot rely on the coercive measures of legitimate states to deter opportunistic behavior—since they lack the backing of legal authority and physical force to punish predatory individuals—what are the mechanisms that enable these unexpected outcomes? Opportunistic behaviour in darknet market transactions can come from platforms or sellers. The first type is platform fraud, which occurs when darknet market administrators carry out an exit scam, closing the markets and disappearing with cryptocurrencies stored in escrow accounts, sometimes worth millions of dollars [39].

## Theoretical framework and hypotheses

Platforms and sellers can adopt various signals to shape buyers’ and sellers ‘behaviour. For instance, it is common for the platform operator to offer its sellers two payment options: centralised via the escrow system, decentralised via Finalise Early Payment (FE) or both, with the decision resting with the sellers. With the escrow system, the seller only receives payment after the buyer has received the item and confirmed it with the platform. This allows the platform administrators to resolve conflicts. However, several factors can make the escrow method less attractive to sellers. First, selling through an escrow account means that payment is less secure, as the transaction amount is deposited in an escrow wallet and depends on the buyer’s confirmation [40]. Moreover, depositing funds in the platform’s wallet increases the risk of loss as law enforcement can authorities seize the platform’s assets [41]. On the other hand, the FE payment method allows the seller to ship goods only after payment is received, reducing the risk of disputes between buyers or loss of funds due to platform seizure. Finally, sellers on the darknet market have a space in which they can describe their offers and provide buyers with all the necessary information. This space is generally not subject to any restrictions, so sellers can use it as they see fit. Similarly, reputation systems can serve as a form of social influence, often implemented through online reviews and ratings. Additionally, as the introduction mentions, sellers can persuade buyers by describing their products. Product information, which includes details and representations of specific items, typically consists of text and photos. To the best of our knowledge, research in this area has not yet analysed the specific characteristics of product information on the darknet market and how these elements contribute to or mitigate buyers’ perception of risk and susceptibility to fraud. Through these forms of influence, platforms and sellers send signals to increase the number of participants and transactions and for sellers to maximise their earnings. Are these signals able to prevent opportunistic behaviour?

### Changing incentive structure through escrow as a deterrent for opportunistic behaviour in dark web transactions

**Fig 1:**
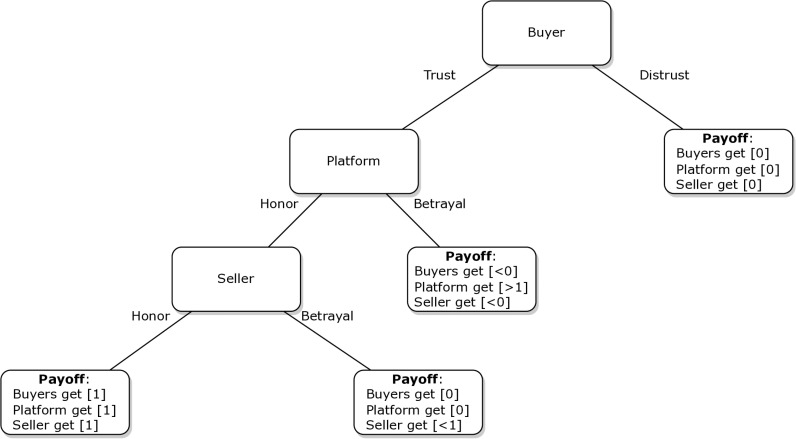
Trust interaction with the escrow system.

Escrow payments can be a costly signal that prevents opportunistic behaviour by sellers as they radically change the structure of incentives in interactions. If we analyse the interaction from the perspective of the trust game, we can identify several possible outcomes. Buyers can choose to cooperate or not (i.e., buy or not buy a product). If they decide not to cooperate (i.e., not to buy), all actors receive a zero payoff (i.e., the seller does not receive the money, and the buyer does not receive the desired product). Alternatively, if the buyer decides to cooperate with the escrow system, they deposit the transaction amount in the platform account, giving the platform two options: to defect or to cooperate. If the platform administrator decides to defect, they will receive a gain greater than 1, while the seller and buyer will receive less than 0. This option is also called an ‘exit scam’. Otherwise, if the platform decides to cooperate, the decision passes to the seller. If the seller cooperates, all the actors receive an amount equal to 1, while if the seller decides to defect, they receive a payoff less than 0, while other actors receive one equal to 0. Thus, the social interaction illustrated in Fig 1 differs significantly from the social interaction of the traditional trust problem.

The escrow system’s main weakness is its centrality in managing transactions. If law enforcement shuts down the market, all the currencies deposited in the platform’s wallet are seized. Furthermore, if a platform decides to close its activity, buyers lose the money they have deposited in escrow. Research has analysed the impact of escrow on sales and concluded that it has a negative impact. The main mechanisms identified so far to explain this effect relate to the price increase associated with the commission that platforms charge for their involvement in dispute resolution [19,35]. However, the introduction of an escrow account serves as a safeguard against opportunistic behaviour. By entrusting payment to a third party, buyers gain assurance that sellers will hold up their end of the deal. Escrow accounts create a structured environment in which funds are released to sellers only after the satisfactory delivery of goods or services. It is, therefore, reasonable to assume that escrow is a costly signal that sellers send out to demonstrate their trustworthiness. In this context, it is plausible to hypothesise that the risk of opportunistic behaviour increases for transactions conducted outside the protective umbrella of an escrow service.

H1: Transactions conducted outside the escrow system are more likely to be scams.

### Changing incentive structure through reputation as a deterrent for opportunistic behaviour in dark web transactions

The most common regulation in digital markets involves the feedback system, a tool that directly enhances cooperation through a diffuse control system [42]. In this system, the buyer rates the seller when a transaction is complete. The feedback system is an essential generator of reputation. It shapes market interactions since buyers could punish the seller through an immaterial sanction that implicitly becomes material. Indeed, many empirical studies have confirmed Shapiro’s reputation theory [43], which states that a bad reputation negatively affects the price and number of sales of a product or service [20,44,45]. In this context, the objective structure of social relations is partially shaped by comparing it to the previously described structure at the beginning of section two, in the absence of a reputation system. Reputation creates an incentive for cooperative behaviour and a disincentive for opportunistic behaviour. In addition, the trustor’s uncertainty is reduced since the trustor can control the trustee’s past behaviour. Buyers can trust sellers’ claims due to the emphasis sellers place on maintaining their reputation [27,44,46–49]. Consequently, the establishment of reputation systems becomes imperative to address issues of distrust and malfeasance in markets [50–53]. In light of these considerations, it is reasonable to assume that when sellers attain a better reputation and are capable of charging premium prices, they do not behave opportunistically to maximise gain.

H2: As the percentage of positive reviews increases, the probability of scams decreases.

### Persuasive ‘cheap talk’ as a tool for fraudulent buyers

In the darknet market, vendors can promote their products within the space provided for item descriptions. The inherent time gap between a buyer placing an order and receiving the product in online transactions makes physically inspecting quality impractical. This poses significant challenges, such as information asymmetry and adverse selection problems on online platforms [47]. To tackle these challenges product description becomes crucial for buyers in assessing a seller’s reliability. This information can be broadly classified into a cheap signal. Costly signals are only produced by sellers committed to delivering high-quality goods, whereas cheap signals are easily falsifiable and do not reliably indicate a seller’s true intentions. The efficacy of a signal is determined by the associated costs and the anticipated benefits it is expected to generate. [20,54]. On the one hand, product descriptions can serve as a signal of trust. Product descriptions can have a direct impact on a person’s ability to participate in the market, because those who cannot describe their products fluently may not be able to communicate effectively. Therefore, a polished product description can serve as a signal of trust. The predominant language used in forums can have a direct impact on an individual’s ability to participate in the marketplace, as those who cannot describe their products fluently may not be able to communicate effectively. Consequently, sellers may seek to assure buyers of their positive intentions through product descriptions. However, such descriptions can be altered by both trustworthy and untrustworthy sellers.Therefore, item descriptions should be regarded as a low-cost signal or ‘cheap talk’ [27], offering insights into product features and shipping details to mitigate adverse selection. Empirical evidence indicates that these product descriptions, even if considered inexpensive signals, exert a notable influence on buyers’ decision-making processes [46,47,49,55]. Empirical research conducted on traditional e-commerce revealed that item descriptions have a significant impact on sales in the e-commerce sector [46], while images and textual content shared by sellers on auction websites wield considerable influence over prices [47]. Correspondingly, other studies [55] discovered that highlighting high shipping and handling costs leads to a reduction in the winning bid prices on platforms like eBay. However, a gap exists in the literature in terms of analysing how these inexpensive signals are connected to opportunistic behaviour. Following Gambetta’s theory [56], we can hypothesise that, given the affordability of such signals, unscrupulous sellers may exploit them to manipulate buyers into trusting them. In these terms, it is reasonable to anticipate that sellers who adopt a positive tone, provide extended item information, and employ a wide range of vocabulary might engage in opportunistic behaviours. Therefore, it is reasonable to hypothesise that:

H3: As the length of the product description increases, the probability or number of scams also increases.
H4: As the linguistic diversity of the product description increases, the probability or number of scams also increase.
H5: As the sentiment score of the product description increases, the probability or number of scams also increases.

### Seller lifespan as a signal against fraud

Most darknet markets indicate the date the sellers started selling on the market. Alphabay market contains this information, as you can see in the attachment. As Axelrod [57] show, the shadow of the future has an impact on increasing trust and deterring opportunistic behaviour. In his influential work [57], Axelrod coined the phrase’the shadow of the future’ to suggest that people often cooperate because they anticipate rewards for cooperation and punishments for non-cooperation and take a longer-term perspective on the situation at hand. While theory allows for the emergence of cooperation in infinitely repeated games, defeat in finite repetitions is rationally inevitable thanks to backward induction from the last to the first round of the game. Accordingly, long-term participation in the market becomes a signal of the concept of the’shadow of the future’, suggesting that expected future interactions influence present behaviour. Similarly, the lifespan of criminal actors on online carding forums, particularly in the form of’long-term participation in the market,’ has been identified as an indicator of trust [29, 144]. Prior research also identified the length of time vendors are active on the sites as a signal of trust [33], showing that seniority is significantly and positively correlated with criminal opportunity. This suggests that longer engagement may improve criminal performance, which indicates reliability. It is, therefore, reasonable to hypothesise that the longer sellers have been in the market, the lower the probability or number of fraud cases. Darknet markets commonly display the seller’s registration date, as Alphabay market proves (S1 Fig). Based on [57]’s concept of the’shadow of the future,’ which assumes that anticipated future interactions influence current behaviour by increasing trust and deterring opportunistic behaviour, it is hypothesized that sellers with longer tenure in the marketplace have a lower likelihood or frequency of fraudulent activity.

H6: As sellers’ lifespan increases, the probability or number of scams decreases.

## Data and methods

### Data

This study focuses on AlphaBay, the largest crypto market to date. As described in previous chapters, this crypto market operated from December 2014 until July 2017, when an international police operation shut it down. This study focuses on AlphaBay for two important reasons. First, AlphaBay’s payment method offers a unique opportunity to assess seller performance to measure the impact on fraud, as the marketplace’s management gave sellers the ability to choose between two primary payment systems to facilitate transactions: finalise early payment and the escrow system. Finally, sellers on the AlphaBay marketplace can describe their products freely and in detail, with minimal content restrictions and the ability to analyse the impact of the product description on sales. When analysing the AlphaBay crypto market, we used a dataset collected by McKenna and Goode [58]. This dataset is part of a research data collection on dark web markets [59], which includes web scraping of several darknet markets. These datasets are available for research purposes. The use of this dataset in this paper complies with the terms and conditions set forth by the creators of the archive [59]. The dataset used in this work includes 114,385 items, 6,033 sellers, and 1,270,000 reviews. The data were collected between 26 January 2017 and 28 January 2017. This protocol ensured that most listings on the AlphaBay platform were included, regardless of whether the associated items were purchased. Notably, the scraping process encountered some limitations, as 1,636 TOR pages were found to be inaccessible, resulting in approximately 700 missing listings. Although this missing data represents only 0.01 percent of all listings, its impact on our results is negligible. We extracted the following vendor-level data from the TOR pages: nickname, number of listings, and lifespan as a vendor on the platform. Moreover, we extracted the following listing-level data: product description, number of sales per listing, origin and destinations of the listed goods, payment method, transaction feedback and comments left by the buyers. We excluded all offers with zero sales from the original dataset, as zero sales make fraud impossible (Data are available in the supporting information S2 File).

### Variables

**Fig 2:**
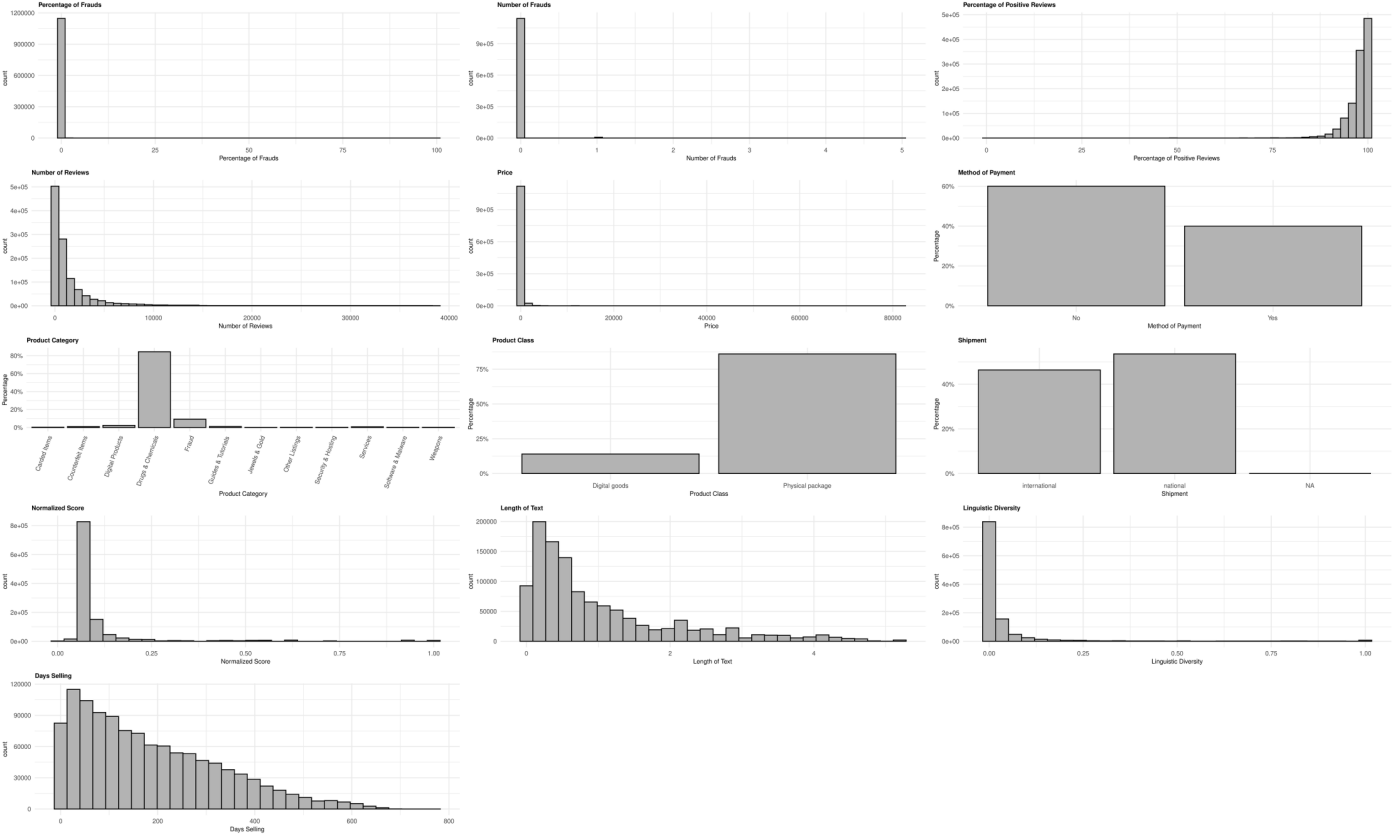
Plot descriptive analysis.

How can the opportunistic behaviour of sellers on darknet markets be measured? Recent research estimated deceptive behaviour by looking for specific words in reviews left by buyers [38]. They found that 60.2 percent of negative feedback was related to delivery problems. In this study, we used a similar approach to estimate the number of fraudulent transactions by identifying words in reviews associated with fraud. We started by counting the 20 most frequent tokens (S2 and S3 Figs) in the negative and neutral feedback. We found that the word’scammer’ appeared, and’scams’ was among the 20 most common words. This result is consistent with other studies, such as [38], underscore that in dark market jargon, individuals who behave dishonestly are frequently labelled as”scammer.” In light of this, we developed a dictionary that includes all words semantically close to”scammer,” such as’fraudster’, ‘con artist’, ‘swindler’, ‘trickster’, ‘deceiver’, ‘cheat’, ‘cheater’, ‘impostor’, ‘charlatan’, ‘hustler’, ‘grifter’, and ‘scam’. These words are detailed in (S1 File). If a word from this list appeared in the review, we classified the review as a scam. For each provider, we counted the number of reviews that could be classified as fraud. Therefore, the dependent variable is the count of the number of scams when the review is published. Our main predictors are the percentage of positive review at the time of the respective review and the payment method as a dummy variable (escrow: ‘yes’ or ‘no’). To test H3 and H4, we created metrics for linguistic diversity in product descriptions and text length [60]. These metrics measure the linguistic diversity used in each offer description by quantifying the unique words within a message [61]. The calculation divides the number of unique words by the total number of words to account for the impact of linguistic diversity on fraud. This addition stems from previous research suggesting that rich text in terms of linguistic diversity better captures users’ attention and increases engagement [62]. To test H4, we introduced the length of the description in relation to the average length of the offers. To do this, we divided the length of each offer description by the average length of all offers on the market. If the resulting value was below one, the description length was below average. Conversely, a value above one meant that the description length was above average [63,64]. For the investigation of H5, we carried out sentiment analysis, a procedure that facilitates the categorisation of sentiment polarity. In the context of a product description, sentiment analysis aims to assign a score to the text. All variable distributions are depicted in Fig 2.

We use the AFINN sentiment lexicon, which assigns scores to text snippets based on a dictionary of sentiment words. These scores range from -5 (negative) to + 5 (positive). Unlike other lexicons, AFINN captures the emotional intensity conveyed by language. By adding the scores of the words categorised in the AFINN lexicon, we calculated a total score for each text analysed [65,60]. As this variable reflects the results of texts of different lengths, we performed min-max Scaling. Min-max scaling is a normalization technique commonly used in data preprocessing to transform numeric variables into a specific range, typically [0, 1] ([66]).


$$Sentiment\;score\;scaled=\frac{Sentiment\;score-\min\;\left(Sentiment\;score\right)}{\max\;\left(Sentiment\;score\right)-\min\;\left(Sentiment\;score\right)}$$

This approach has already been adopted to standardize product descriptions in the darknet market, as seen in [60]. To test H6 as a predictor, we took the number of days since the vendor’s launch at the time the review was completed. Other control variables were included in the model: the price of the product, the type of product (‘physical package’ or ‘digital goods’), the type of shipping (international or domestic, with a value of ‘international’) and finally, the product category. Product categories included ‘counterfeit items’, ‘digital products’, ‘drugs and chemicals’, ‘fraud, guides and tutorials’, ‘jewels’, ‘other listings’, ‘security and hosting’, ‘services, software and malware’ and ‘weapons. These product classifications were determined by the categorisation system used by the Alphabay administrators. An overview of descriptive statistics is presented Table 1 (For the generation of the table, please refer to the S3 File).

**Table 1:** Descriptive statistics.

Variable	Mean	Sd	Median	Min	Max
Percentage of positive reviews	97.7	3.34	98.7	0	100
Number of frauds	0.01	0.09	0	0	5
Percentage of frauds	0	0.01	0	0	1
Days selling	184	145	151	0	769
Price	149	669	48	2.01	82000
Number of reviews	1866	4320	527	1	38709
Length of text	1.01	1.05	0.59	0.01	5.25
Linguistic diversity	0.04	0.13	0.01	0	1
Sentiment score	0.11	0.13	0.07	0	1
**Method of Payment**	**N**	**%**			
Escrow	688,911	60.02			
Finalize early payment	458,857	39.98			
**Product Category**	**N**	**%**			
Carded Items	3,313	0.29			
Counterfeit Items	10,552	0.92			
Digital Products	26,271	2.29			
Drugs & Chemicals	968,240	84.36			
Fraud	105,959	9.23			
Guides & Tutorials	12,729	1.11			
Jewels & Gold	1,651	0.14			
Other Listings	2,581	0.22			
Security & Hosting	2,272	0.20			
Services	8,456	0.74			
Software & Malware	3,201	0.28			
Weapons	2,543	0.22			
**Product Class**	**N**	**%**			
Digital goods	160,887	14.02			
Physical goods	986,881	85.98			
**Shipment**	**N**	**%**			
National	615,037	53.59			
International	532,729	46.41			
Reviews				1,147,768	

### Model estimation

We used a generalised additive model (GAM), allowing for both linear and non-linear relationships between the predictors and the dependent variable. The GAM model explains the dependent variable as an additive combination of parametric and non-parametric functions of the explanatory variable. This model offers two main advantages over ordinary linear regression. First, because the functions generated by non-parametric functions can take on a large variety of shapes, GAM allows uncovering non-linear relationships that optimally adjust the outcome to the predictors and that can condition such relations on covariates. In addition, the GAM model allows residual distribution to follow other than the normality assumption by choosing among different families [67]. Many researchers prefer this model as an alternative to polynomial regression because it does not require specifying the degree of the polynomial, thereby reducing the risk of under- and over-smoothness. By relying on smooth functions of covariates, which penalise unnecessary complexity [68], the GAM model avoids both over-smoothness and under-smoothness. Flexible smooths are created from many smaller functions called basis functions. Each smooth is the sum of a number of basic functions, and each basis function is multiplied by a coefficient, each of which is a parameter in the model. The smoothness of the function is summarised by degrees of freedom. The mathematical form of the GAMS model can be represented as follows:


$$M1:log(E[Y_{i}])=\;\beta_{0}+\;f_{1}(x_{i1})+\;f_{2}(x_{i2})+\;\beta_{3}x_{i3}+\;f_{4}(x_{i4})+\;\beta_{5}x_{i5}+\;\beta_{6}x_{i6}+\;\beta_{7}x_{i7}+\;\beta_{8}x_{i8})$$ (1)

Predictors for Model (1):

- *x*_*i*1_: Percentage of positive reviews (smooth term *f*_1_)
- *x*_*i*2_: Number of reviews (smooth term *f*_2_)
- *x*_*i*3_: Price (linear term with coefficient *β*_3_)
- *x*_*i*4_: Days Selling (smooth term *f*_4_)
- *x*_*i*5_: Product Category (categorical variable with coefficient *β*_5_)
- *x*_*i*6_: Product class (categorical variable with coefficient *β*_6_)
- *x*_*i*7_: Escrow (categorical variable with coefficient *β*_7_)
- *x*_*i*8_: International shipment (smooth term *f*_8_)

$$\begin{array}{l} M2:log(E[Y_{i}])=\\ \;\beta_{0}+\;f_{1}(x_{i1})+\;f_{2}(x_{i2})+\;\beta_{3}x_{i3}+\;f_{4}(x_{i4})+\;\beta_{5}x_{i5}+\;\beta_{6}x_{i6}+\;\beta_{7}x_{i7}+\;\beta_{8}x_{i8}+\beta_{9}x_{i9}+\beta_{10}x_{i10}\;+\;f_{11}(x_{i11}) \end{array}$$ (2)

Predictors for Model (2):

- *x*_*i*1_: Percentage of positive reviews (smooth term *f*_1_)
- *x*_*i*2_: Number of reviews (smooth term *f*_2_)
- *x*_*i*3_: Price (linear term with coefficient *β*_3_)
- *x*_*i*4_: Days selling (smooth term *f*_4_)
- *x*_*i*5_: Product category (categorical variable with coefficient *β*_5_)
- *x*_*i*6_: Product class (categorical variable with coefficient *β*_6_)
- *x*_*i*7_: Escrow (categorical variable with coefficient *β*_7_)
- *x*_*i*8_: International Shipment (smooth term *f*_8_)
- *x*_*i*9_: Length of text (linear term with coefficient *β*_9_)
- *x*_*i*10_: Linguistic diversity (linear term with coefficient *β*_1_0)
- *x*_*i*11_: Sentiment score (linear term with coefficient *β*_11_)

## Results

The first section of Table 2 gives an overview of the fitted models (Refer to the S4 file for the generation of GAM Models in R). The first row indicates the dependent variable for all models, which results from the hypotheses made in the theoretical section. The term ‘family’ refers to the error distribution used in the model. In this particular model, we have chosen the Tweedie distribution.

**Table 2:** GAM Models M1 and M2.

Model Components	M1	M2
**Dependent variable**	Percentage of frauds	Percentage of frauds
**Family**	Tweedie	Tweedie
	(p = 1.597)	(p = 1.594)
**Link function**	log	log
**Variable**	**Estimate**	**Estimate**
Intercept	7.93***(0.29)	8.07***(0.29)
Price	0.00*(0.00)	0.00(0.00)
**Method of Payment**		
Escrow	Reference	Reference
Finalize early payment	0.23***(0.04)	0.24***(0.04)
**Product category**		
Carded Items	Reference	Reference
Counterfeit items	0.34*(0.33)	0.32**(0.33)
Digital products	0.06(0.32)	0.12(0.32)
Drugs & chemicals	0.49(0.32)	0.46(0.32)
Fraud	0.05(0.29)	0.05(0.29)
Guides & tutorials	0.37(0.32)	0.42(0.32)
Jewels & gold	0.23(0.56)	0.26(0.56)
Other listings	0.58(0.51)	0.57(0.49)
Security & hosting	0.63(0.42)	0.57(0.41)
Services	0.66*(0.33)	0.53(0.33)
Software & malware	0.35(0.38)	0.53(0.38)
Weapons	0.14(0.46)	0.38(0.46)
**Product class**		
Product class (Digital goods)	Reference	Reference
Product class (Physical package)	0.40(0.21)	0.34(0.21)
**Shipment**		
National shipment	Reference	Reference
International shipment	0.11**(0.04)	0.10**(0.04)
Sentiment score		0.89***(0.19)
Linguistic diversity		1.55***(0.13)
Length of text		0.03(0.02)
**Smooth variable EDF**		
Percentage of positive reviews	8.95^***^	8.95^***^
Number of reviews	8.18^***^	8.10^***^
Days selling	6.49^***^	5.71^***^
R-sq.(adj)	0.18	0.185
Deviance explained	61.9%	62.3%
AIC	74877	74723
BIC	75387	75260
REML	37495	37417

*Notes:* Standard error in parentheses; (

*** P 0.001,

** P 0.01,

* P 0.05) This choice is particularly important when dealing with data exhibiting characteristics such as excess zeros.

**Fig 3:**
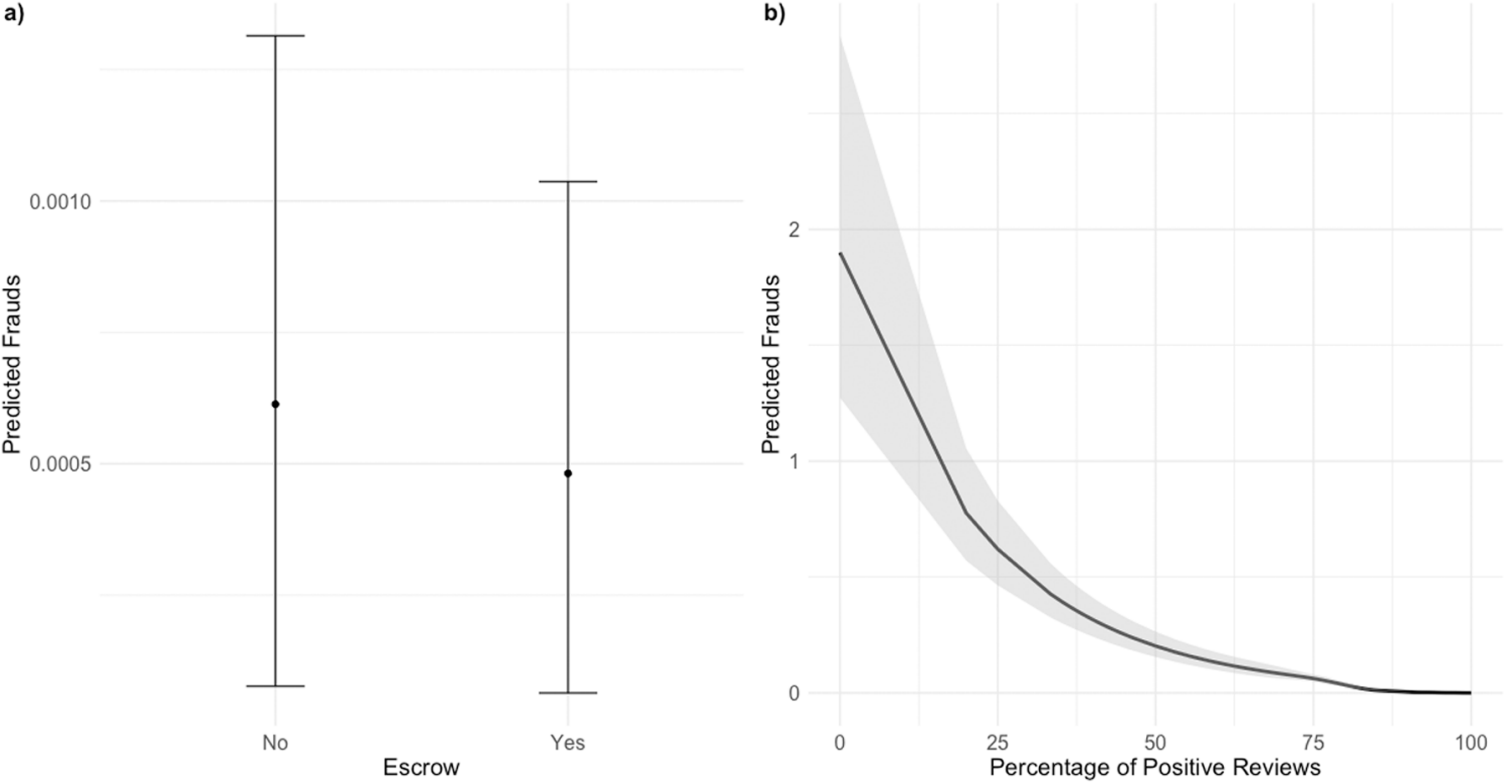
Results of generalised additive models for the estimated impact of escrow (a) and percentage of positive reviews (b) on fraud. Note: In this Fig (a) represents the estimated impact of escrow on fraud, while (b) represents the estimated impact of the percentage of positive reviews on fraud, as analysed through GAMs. The plot displays the predicted values and their corresponding confidence intervals, offering insights into the relationship between escrow, percentage of positive reviews and fraud cases.

Initially, we fitted model M1 without the inclusion of text analysis variables. Subsequently, we incorporated text predictors into the model, resulting in model M2. The additional variables introduced in M2 comprised sentiment score, linguistic diversity, and text length. The objective was to compare the performance of the models. When assessing goodness-of-fit parameters between M1 and M2, we observed improvements with the addition of the text variables. Explained variance increased from 61.9 percent in M1 to 62.3 percent in M2. Furthermore, AIC decreased from 74,877 to 74,723, and BIC decreased from 75,387 to 75,260 in model M2. These enhancements in goodness of fit suggest that models with the inclusion of hypothesis-based predictors, particularly text variables, offer a more concise and accurate representation of the data compared to the initial models, which lacked text predictors. Models 1 and 2 provide empirical support for the hypothesis presented in the theoretical section. The coefficients shown in Table 2 indicate the expected change in the logarithmic number of fraud cases, assuming all other predictors remain constant. In both models, the coefficient associated with the escrow account is consistently significant and negative. This indicates that transactions processed via an escrow payment are associated with a lower probability of fraud. Specifically, in Model 1, the expected number of fraud cases decreases by an estimated -20.69 percent for transactions processed via escrow. The situation is similar in Model 2, which includes text variables. The presence of an escrow account is associated with an estimated decrease in the expected percentage of fraud cases by approximately -21.67 percent. This proportional change is calculated using the formula: Proportional change =  exp(coefficient) – 1 *  100 provide evidence for hypothesis 1. Moving to hypothesis two, we observe a significant effect in both models. A significant EDF means that it is impossible to draw a horizontal line within a 95 percent confidence interval, indicating a non-linear relationship between the percentage of reputation and fraud cases. Fig 3, which represents Model 2 (plot b), shows a general inverse relationship: an increase in the percentage of positive reviews correlates with a decrease in the expected number of fraud cases.

**Fig 4:**
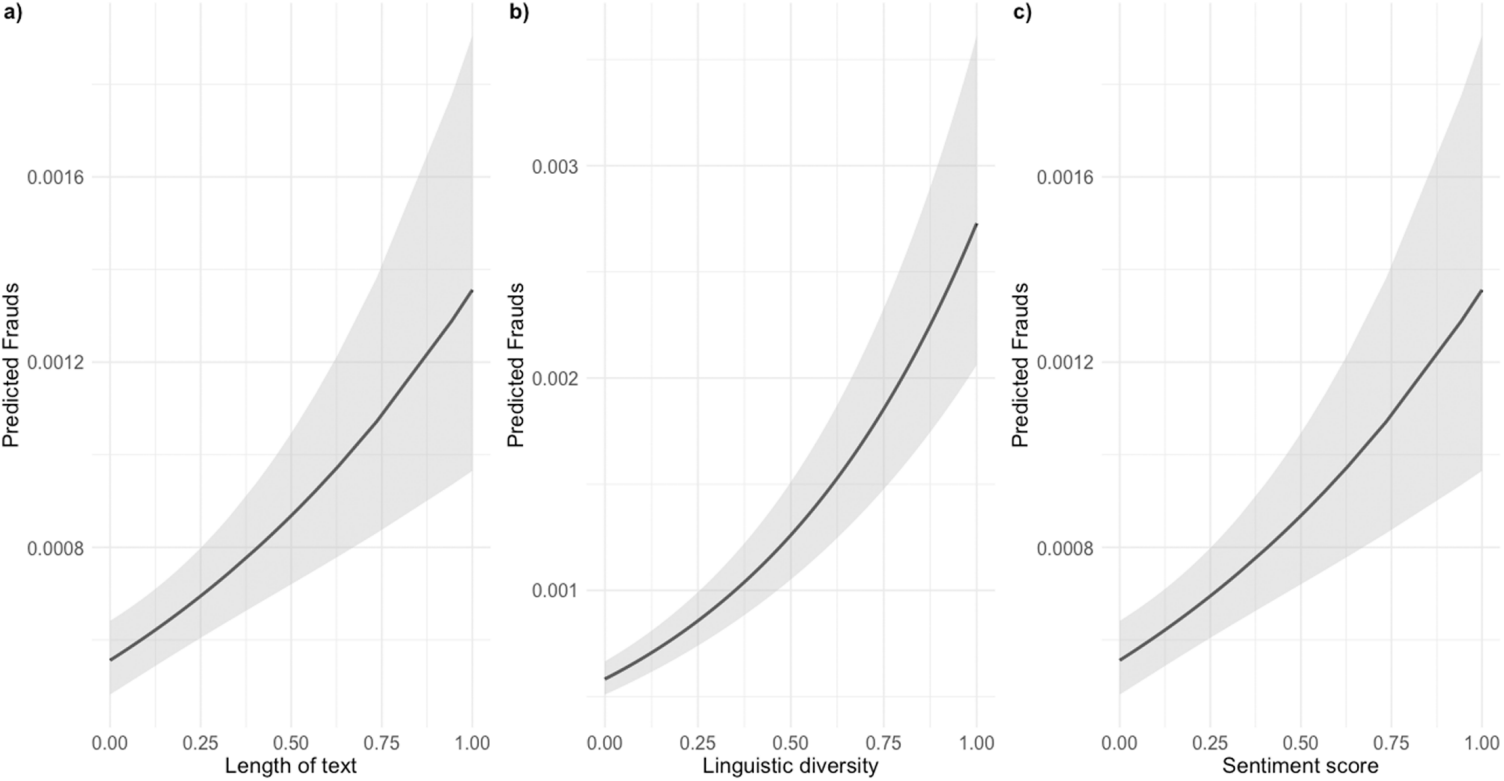
Results of generalised additive models for the estimated impact of (a) length of text of item offers, (b) linguistic diversity of text of item offers, and (c) sentiment score of text of item offers on fraud cases. Note: In this Fig 4, (a) represents the estimated impact of the length of text of item offers, (b) represents the estimated impact of the linguistic diversity of the text of item offers on fraud cases, while (c) represents the estimated impact of sentiment score of the text of item offers on fraud cases as analysed through GAMs.

Regarding the text variables, linguistic diversity and sentiment score have positive coefficients. This indicates that an increase in these variables is associated with an increase in the logarithmic number of fraud cases. Specifically, the coefficient is 0.03 in Model 2 for the length of offer text, 1.55 in Model 2 for linguistic diversity, and finally, 0.89 for the sentiment score of product description in Model 2, respectively (see Fig 4).

The plot displays the predicted values and their corresponding confidence intervals, offering insights into the relationship between the length of the text of item offers, the linguistic diversity of the text of item offers, and the sentiment score of the text of item offers and fraud cases.

These results provide evidence for H4 and H5 and show that sellers who invest in writing complex item listings in terms of vocabulary variety and use of positive words tend to be more deceptive.

While H3 is only partially confirmed, there is some evidence suggesting an association between the length of the text and an increase in fraud, as indicated by the coefficient (Table 2). However, it’s worth noting that the p-value associated with this coefficient is 0.063, which falls just outside the conventional significance level of 0.05.

**Fig 5:**
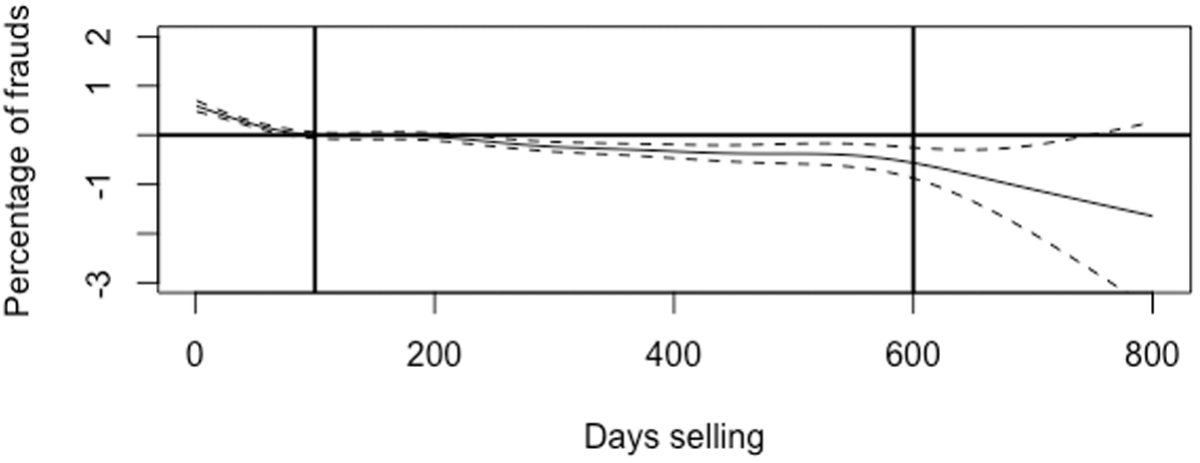
Results of generalized additive models for the estimated impact of day selling on fraud cases. Note: The plot illustrates the nonlinear relationship between the number of days the product has been on the market and frauds.

Moving to H6 in Table 2, we observe a non-linear relationship between daily sales and fraud with statistical significance. In particular, the analysis of the smooth terms in M2 shows a non-linear relationship between daily sales and fraud with a confidence interval of 99 per cent. Fig 4 shows plots of the smooth terms indicating that daily sales show an irregular and negative trend with three different patterns: The first pattern extends from 0 to 100 days where the relationship between daily sales and fraud is initially positive but dramatically decreases, the second patterns extends from 100 to 600 days where the relationship decreases slightly and becomes negative, and the third phase occurs after 600 days where the relationship decreases dramatically; however, confidence intervals above this threshold tend to widen, potentially reducing the reliability of the observed trends (Fig 5). Despite this irregularity, there is a general trend that fraud decreases as the lifespan of the seller increases, which empirically proves that the lifespan of the seller is a reliable signal of the seller’s credibility.

## Conclusion

In the darknet, the problem of social order increases dramatically, as there is no legitimate authority to punish and prevent opportunistic behaviour. However, previous studies show that the market is characterised by a high level of trust [20] with a relatively high percentage of positive reviews [38]. Even in these studies, this result is confirmed as 97.7 percent of reviews are positive, and frauds are extremely rare. How is this possible? How can an unregulated environment such as the dark internet mitigate frauds so radically? Unlike the various studies on darknet platforms that focus on the impact of reputation on sales [20,45,69,70], this work by adopting the signalling theory has examined the factors that influence the number of fraud cases. The results provide four important pieces of evidence. First and second, escrow and reputations are costly and reliable signals that significantly reduce the number of fraud cases. By contrast, ‘cheap talk’, as expressed by the length of text in article descriptions, linguistic variety and sentiment value are unreliable, which is associated with an increase in fraud, holding everything constant. Lastly seller long-term participation in the market is associated with a decrease of frauds. How can these results be explained? Costly signals such as escrow services provide a secure intermediary for transactions and ensure that funds are held until both parties fulfil their obligations. This discourages fraudulent behaviour, as the risk of losing money is higher for sellers who fail to live up to buyers’ trust. In addition, a good reputation acts as a deterrent to potential fraudsters. A good reputation leads to higher sales and premium prices, but it is difficult to build and easy to lose, increasing the cost of opportunistic behaviour. Therefore, reputation becomes a reliable signal which favours the social order of the market reducing frauds. Additionally, as seller seniority is correlated with criminal opportunity [30], it becomes a costly signal that sellers emit about their reliability. However, the product description is an ambiguous signal. On the one hand, studies on both legal and illegal online markets show that product information acts as a risk-reducing signal and reduces adverse selection [21,60]. On the other hand, fraudsters on darknet markets can use sophisticated product descriptions or’cheap talk’ to defraud their victims. By creating persuasive item descriptions, using different language styles and manipulating the sentiment of a product description, sellers can try to appear more legitimate and trustworthy and gain the opportunity to abuse buyers’ trust. In summary, the observed results suggest combining secure transaction mechanisms (escrow) and deterrent reputation features effectively reduces fraud. In this context, however, the role of reputation becomes particularly influential. On the other hand, the increase in fraud cases related to sophisticated ‘cheap talk’ emphasises that fraudsters can use manipulative communication strategies to deceive potential victims. In short, in the context of darknet markets, costly signals such as a positive reputation, seller seniority and escrow services require significant investment or effort from sellers, making opportunistic behaviour less convenient compared to cheap signals such as detailed product descriptions, which are easier to manipulate or falsify. In a scenario where online transactions have increased dramatically; these outcomes have implications even for online commerce beyond the darknet. While reputation systems are well-known for reducing fraud, escrow payments are not widely adopted. This system could be especially beneficial for new sellers entering the market who lack sufficient reputation. In such cases, costly signals like escrow could be a tool to mitigate fraud in the online context and allow sellers to enter the market. At the same time, what we learn from these studies is that fraudulent sellers often adopt a positive tone and persuasive product descriptions. Considering these results, future campaigns against fraud in the online context should stress that buyers should be sceptical of descriptions that use enthusiastic language and do not provide costly signals of guarantee, such as protection in the method of payment and reputation. Although this study provides valuable insights, certain limitations must be acknowledged. Since the dataset is cross-sectional, it does not account for temporal changes, making it difficult to analyze how the phenomenon evolves over time. This limitation is partly derived from the nature of darknet environments, which are highly dynamic and unpredictable, posing challenges for conducting longitudinal research that could offer a more in-depth understanding of fraud trend.

## Supporting information

S1 Fig Example of auction listing. (TIF)

S2 Fig Top 20 most frequent words. Plot of the 20 most frequent words in negative and neutral reviews.
(TIF)

S3 Fig Non-linear relationship M2. Plot of the smooth terms, illustrating the effects of the Smooth variables on fraud for model 2.
(TIF)

S1 File Frauds words. These are the terms used to identify in reviews where buyers reported a fraud.
(TIF)

S2 File Data. (ZIP)

S3 File Tables. (PDF)

S4 File Gam model. (PDF)

## References

[1] Aspers P. Knowledge and valuation in markets. Theory and Society. 2009;38:111–31.

[2] Beckert J. The social order of markets. Theory and Society. 2009;38:245–69.

[3] Tzanetakis M. Informal governance on cryptomarkets for illicit drugs. In: Governance beyond the law: The immoral, the illegal, the criminal. 2019. Palgrave Macmillan. p. 343–61.

[4] Tzanetakis M, Kamphausen G, Werse B, von Laufenberg R. The transparency paradox. Building trust, resolving disputes and optimising logistics on conventional and online drugs markets. Int J Drug Policy. 2016;35:58–68. doi: 10.1016/j.drugpo.2015.12.010 26809972

[5] Beckert J, Wehinger F. In the shadow: illegal markets and economic sociology. Socio-Economic Review. 2012;11(1):5–30. doi: 10.1093/ser/mws020

[6] Kim G, Koo H. The causal relationship between risk and trust in the online marketplace: A bidirectional perspective. Computers in Human Behavior. 2016;55:1020–9.

[7] Chen JV, Yen DC, Kuo W-R, Capistrano EPS. The antecedents of purchase and re-purchase intentions of online auction consumers. Computers in Human Behavior. 2016;54:186–96.

[8] Gambetta D. Codes of the underworld: How criminals communicate. Princeton University Press; 2009.

[9] Luhmann N. Familiarity, confidence, trust: Problems and alternatives. In Diego Gambetta, editor, Trust: Making and Breaking Cooperative Relations. Blackwell, Oxford. 1988. p. 94–107.

[10] Martin J. Drugs on the Dark Net: How Cryptomarkets are Transforming the Global Trade in Illicit Drugs. Springer, 2014.

[11] Demant J, Munksgaard R, Décary-Hétu D, Aldridge J. Going local on a global platform: A critical analysis of the transformative potential of cryptomarkets for organized illicit drug crime. International Criminal Justice Review. 2018;28(3):255–74.

[12] Soska K, Christin N. Measuring the longitudinal evolution of the online anonymous marketplace ecosystem. In 24th USENIX security symposium (USENIX security 15). 2015. p. 33–48.

[13] Rochet J-C, Tirole J. Platform competition in two-sided markets. Journal of the European Economic Association. 2003;1(4):990–1029.

[14] Tzanetakis M. Comparing cryptomarkets for drugs. A characterisation of sellers and buyers over time. Int J Drug Policy. 2018;56:176–86. doi: 10.1016/j.drugpo.2018.01.022 29449105

[15] Espinosa R. Scamming and the reputation of drug dealers on Darknet Markets. International Journal of Industrial Organization. 2019;67:102523. doi: 10.1016/j.ijindorg.2019.102523

[16] Macanovic A, Przepiorka W. The moral embeddedness of cryptomarkets: text mining feedback on economic exchanges on the dark web. Socio-Economic Review. 2023:mwad069.

[17] Décary-Hétu D, Quessy-Doré O. Are repeat buyers in cryptomarkets loyal customers? Repeat business between dyads of cryptomarket vendors and users. American Behavioral Scientist. 2017;61(11):1341–57.

[18] Norbutas L, Ruiter S, Corten R. Believe it when you see it: Dyadic embeddedness and reputation effects on trust in cryptomarkets for illegal drugs. Social Networks. 2020;63(1):150–61.

[19] Munksgaard R. Building a case for trust: reputation, institutional regulation and social ties in online drug markets. Global Crime. 2023;24(1):49–72.

[20] Przepiorka W, Norbutas L, Corten R. Order without law: Reputation promotes cooperation in a cryptomarket for illegal drugs. European Sociological Review. 2017;33(6):752–64.

[21] Selmar M, Verhagen T. Reducing consumer risk in electronic marketplaces: The signaling role of product and seller information. Computers in Human Behavior. 2018;86:205–17.

[22] Li W, Wang L, Liu F, Bae S. How to persuade an online gamer to give up cheating? uniting elaboration likelihood model and signaling theory. Computers in Human Behavior. 2023;96(1):149–62.

[23] Gregory CK, Meade AW, Thompson LF. Understanding internet recruitment via signaling theory and the elaboration likelihood model. Computers in Human Behavior. 2013;29(5):1949–59.

[24] Chang R, Wei X, Zhang X, Xiong H, Zhu H. How recommendation letters affect career mobility: Evidence from a social networking sites linkedin. Computers in Human Behavior. 2024;152:108084.

[25] Feine J, Morana S, Gnewuch U. Measuring service encounter satisfaction with customer service chatbots using sentiment analysis. Journal of Customer Service. 2019;10(2):123–45. doi: 10.1234/jcs.2019.001

[26] Przepiorka W, Berger J. Signaling theory evolving: Signals and signs of trustworthiness in social exchange. Social dilemmas, institutions and the evolution of cooperation. 2017:373–92.

[27] Kas J, Corten R, van de Rijt A. Trust, reputation, and the value of promises in online auctions of used goods. Rationality and Society. 2023;10434631231170342.

[28] Décary-Hétu D, Paquet-Clouston M, Aldridge J. Going international? Risk taking by cryptomarket drug vendors. Int J Drug Policy. 2016;35:69–76. doi: 10.1016/j.drugpo.2016.06.003 27453145

[29] Holt TJ, Smirnova O, Hutchings A. Examining signals of trust in criminal markets online. Journal of Cybersecurity. 2016;2(2):137–45.

[30] Décary-Hétu D, Leppänen A. Criminals and signals: An assessment of criminal performance in the carding underworld. Security Journal. 2016;29:442–60.

[31] Andrei F, Veltri GA. Status Spill-Over in Cryptomarket for Illegal Goods. Social Science Computer Review. 2024;0(0): null. doi: 10.1177/08944393241286339

[32] Hardin R. The street-level epistemology of trust. Politics & Society. 1993;21(4):505–29.

[33] Décary-Hétu D, Laferriére D. Discrediting vendors in online criminal markets. Disrupting criminal networks: Network analysis in crime prevention. 2015. p. 129–52.

[34] Rompf SA. Trust and rationality: an integrative framework for trust research. Springer, 2014.

[35] Filippo Andrei F, Barrera D, Krakowski K, Sulis E. Trust intermediary in a cryptomarket for illegal drugs. European Sociological Review. 2023:jcad020.

[36] Nooteboom B. Forms, sources and processes of trust. In: Bachmann R, Zaheer A, editors, Handbook of Trust Research. Edward Elgar, 2006. p. 247–63.

[37] Coleman JS. Foundations of social theory. Harvard University Press; 1994.

[38] Venkataraman B, Linacre R, Machin S. The economic functioning of online drugs markets. Journal of Economic Behavior & Organization. 2019;159:426–41.

[39] Van Buskirk J, Bruno R, Dobbins T, Breen C, Burns L, Naicker S, et al. The recovery of online drug markets following law enforcement and other disruptions. Drug Alcohol Depend. 2017;173:159–62. doi: 10.1016/j.drugalcdep.2017.01.004 28259089

[40] Tzanetakis M. Social order of anonymous digital markets: Towards an economic sociology of cryptomarkets. Place, space and time in european drug use, markets and policy. 2018;61–80.

[41] Childs A, Coomber R, Bull M, Barratt MJ. Evolving and diversifying selling practices on drug cryptomarkets: An exploration of off-platform “direct dealing”. Journal of Drug Issues. 2020;50(2):173–90.

[42] Matzat U. A theory of relational signals in online groups. New Media & Society. 2009;11(3):375–94.

[43] Shapiro C. Consumer information, product quality, and seller reputation. The Bell Journal of Economics. 1982;20–35.

[44] Ba S, Pavlou PA. Evidence of the effect of trust building technology in electronic markets: Price premiums and buyer behavior. MIS Quarterly. 2002;26(3):243–68. doi: 10.2307/4132312

[45] Hardy RA, Norgaard JR. Reputation in the internet black market: an empirical and theoretical analysis of the deep web. Journal of Institutional Economics. 2016;12(3):515–39.

[46] Houser D, Wooders J. Reputation in auctions: Theory, and evidence from ebay. Journal of Economics & Management Strategy. 2006;15(2):353–69.

[47] Lewis G. Asymmetric information, adverse selection and online disclosure: The case of ebay motors. American Economic Review. 2011;101(4):1535–46.

[48] Melnik MI, Alm J. Seller reputation, information signals, and prices for heterogeneous coins on ebay. Southern Economic Journal. 2005;72(2):305–28.

[49] Snijders C, Zijdeman R. Reputation and internet auctions: ebay and beyond. Analyse & Kritik. 2004;26(1):158–84.

[50] Cook KS, Hardin R, Levi M. Cooperation without trust? Russell Sage Foundation; 2005.

[51] Greif A. Reputation and coalitions in medieval trade: evidence on the maghribi traders. The Journal of Economic History. 1989;49(4):857–82.

[52] Swedberg R. Joseph A. Schumpeter: The economics and sociology of capitalism. Princeton University Press, 2020.

[53] Zucker LG. Production of trust: Institutional sources of economic structure, 1840–1920. Research in organizational behavior. 1986.

[54] Gambetta D, Szekely A. Signs and (counter) signals of trustworthiness. Journal of Economic Behavior & Organization. 2014;106:281–97.

[55] McDonald CG, Slawson VC Jr. Reputation in an internet auction market. Economic Inquiry. 2002;40(4):633–50. doi: 10.1093/ei/40.4.633

[56] Gambetta D. Signaling. In P. Hedstrom and P. Bearman, editors, The Oxford Handbook of Analytical Sociology. Oxford University Press; 2009. p. 168–94.

[57] Axelrod R, Hamilton WD. The evolution of cooperation. Science. 1981;211(4489):1390–6. doi: 10.1126/science.7466396 7466396

[58] Michael M, Sigi G. Darknet market archives, 2017. Retrieved from: https://www.gwern.net/DNM-archives

[59] Branwen G, Christin N, D´ecary-H´etu D, Munksgaard Andersen R, StExo, El Presidente, et al. Dark net market archives, 2011-2015. https://gwern.net/dnm-archive, July 2015 [Accessed: 2023-11-01].

[60] Andrei F, Veltri GA. Social influence in the darknet market: The impact of product descriptions on cocaine sales. Int J Drug Policy. 2024;124:104328. doi: 10.1016/j.drugpo.2024.104328 38245917

[61] Huffaker D. Dimensions of Leadership and Social Influence in Online Communities. Human Communication Research. 2010;36(4): 593–617. doi: 10.1111/j.1468-2958.2010.01390.x

[62] Berthold MR, Sudweeks F, Newton S, Coyne RD. It makes sense: Using an autoassociative neural network to explore typicality in computer mediated discussions. 1998.

[63] Huffaker D. Dimensions of leadership and social influence in online communities. Human Communication Research. 2010;36(4):593–617.

[64] Spitters M, Klaver F, Koot G, Van Staalduinen M. Authorship analysis on dark marketplace forums. In: 2015 European Intelligence and Security Informatics Conference. IEEE; 2015. p. 1–8.

[65] Nielsen FA. A new anew: Evaluation of a word list for sentiment analysis in microblogs. arXiv preprint arXiv:1103.2903. 2011.

[66] James G, Witten D, Hastie T, Tibshirani R. An introduction to statistical learning. Springer; 2013;112.

[67] Hastie TJ. Generalized additive models. In: Statistical models in S. Routledge, 2017. p. 249–307.

[68] Wood N. Generalized additive models: an introduction with R. CRC Press, 2017.

[69] Diekmann A, Jann B, Przepiorka W, Wehrli S. Reputation formation and the evolution of cooperation in anonymous online markets. American sociological review. 2014;79(1):65–85.

[70] Janetos N, Tilly J. Reputation dynamics in a market for illicit drugs. arXiv preprint arXiv:1703.01937. 2017.

