# Supplementary figures and images for: Signalling strategies and opportunistic behaviour: Insights from dark-net markets
Source: PLoS One. 2025 Mar 18;20(3):e0319794. doi: 10.1371/journal.pone.0319794 (PMC11918442; doi:10.1371/journal.pone.0319794)

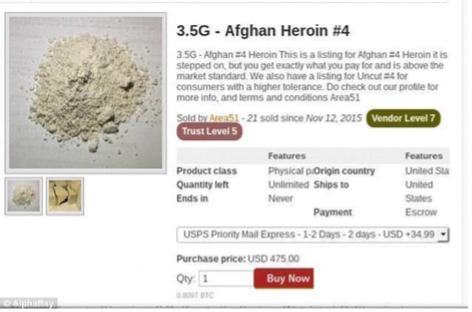

Supplement: S1 Fig — (TIF) [file pone.0319794.s001.tif]

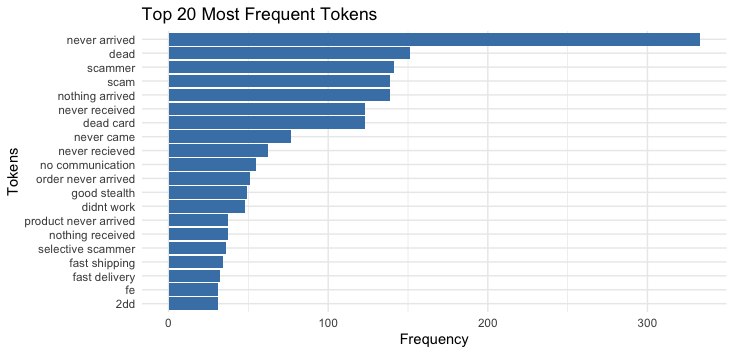

Supplement: S2 Fig — Plot of the 20 most frequent words in negative and neutral reviews. (TIF) [file pone.0319794.s002.tif]

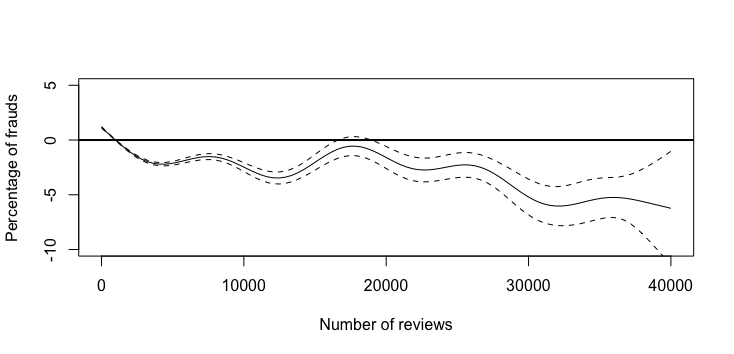

Supplement: S3 Fig — Plot of the smooth terms, illustrating the effects of the Smooth variables on fraud for model 2. (TIF) [file pone.0319794.s003.tif]

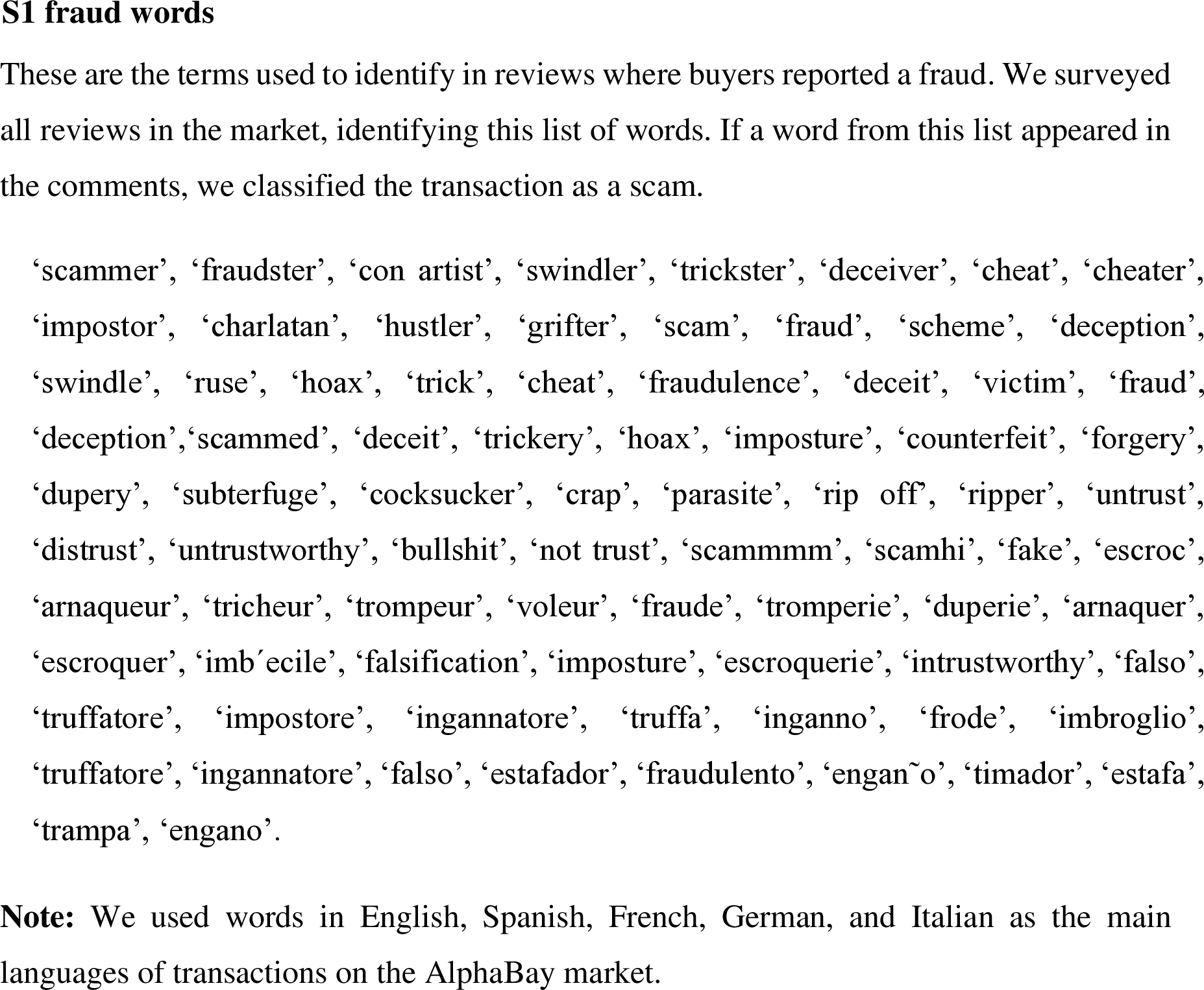

Supplement: S1 File — These are the terms used to identify in reviews where buyers reported a fraud. (TIF) [file pone.0319794.s004.tif]
