# Supplementary material for: Signalling strategies and opportunistic behaviour: Insights from dark-net markets
Source: PLoS One. 2025 Mar 18;20(3):e0319794. doi: 10.1371/journal.pone.0319794 (PMC11918442; doi:10.1371/journal.pone.0319794)
Supplement: S3 File — (PDF) [file pone.0319794.s006.pdf]

```r
library(readr)
data <- read_csv("S1 data.csv")



#table1----
#Numeric variables
library(dplyr)    # For data manipulation
library(knitr)    # For creating tables
library(kableExtra) # For enhanced table formatting

# Define the variables to summarize
variables  <-  c("percentage_of_frauds",  "number_of_frauds",  "perc_positive_reviews", "n_of_reviews", "price", "normalized_score", "length_of_text", "linguistic_diversity", "days_selling")

# Create a summary table with rounded values
summary_table <- data.frame(
  Variable = variables,
  Mean = sapply(variables, function(x) round(mean(data[[x]], na.rm = TRUE), 2)),
  SD = sapply(variables, function(x) round(sd(data[[x]], na.rm = TRUE), 2)),
  Median = sapply(variables, function(x) round(median(data[[x]], na.rm = TRUE), 2)),
  Min = sapply(variables, function(x) round(min(data[[x]], na.rm = TRUE), 2)),
  Max = sapply(variables, function(x) round(max(data[[x]], na.rm = TRUE), 2))
)
summary_table
#categorical variable
# Load necessary library
library(dplyr)
# Function to create summary table for a given categorical variable
create_summary <- function(variable, name) {
  counts <- table(variable)
  percentages <- prop.table(counts) * 100
  data.frame(
    Variable = name,
    Category = names(counts),
    Count = sprintf("%.2f", as.numeric(counts)),
    Percentage = sprintf("%.2f", as.numeric(percentages))
  )
}
# Create summary for each variable
payment_summary <- create_summary(data$method_of_payment, "Method of Payment")
class_summary <- create_summary(data$product_class, "Product Class")
category_summary <- create_summary(data$product_category, "Product Category")
shipment_summary <- create_summary(data$shipment, "Shipment")
# Combine all summaries into a single data frame
categorical_variable  <-  bind_rows(payment_summary,  class_summary,  category_summary, shipment_summary)
# Print the combined summary table
print(categorical_variable )
```

```
#table2----
gam_model1 <- readRDS("gam_model1.rds")
gam_model2 <- readRDS("gam_model2.rds")
options(digits = 3)

library(mgcv)


# Summary for Model 1
summary_gam_model1 <- summary(gam_model1)
M1_rounded_coeffs <- round(coef(gam_model1), 2)
M1_standard_errors <- round(summary_gam_model1$se, 2)

M1_AIC <- AIC(gam_model1)
M1_BIC <- BIC(gam_model1)

# Summary for Model 2
summary_gam_model2 <- summary(gam_model2)
M2_rounded_coeffs <- round(coef(gam_model2), 2)
M2_standard_errors <- round(summary_gam_model2$se, 2)


M2_AIC <- AIC(gam_model2)
M2_BIC <- BIC(gam_model2)

# Create a table for Model 1
table_model1 <- data.frame(
  Term = names(M1_rounded_coeffs),
  Coefficient = M1_rounded_coeffs,
  Std_Error = M1_standard_errors
)

# Create a table for Model 2
table_model2 <- data.frame(
  Term = names(M2_rounded_coeffs),
  Coefficient = M2_rounded_coeffs,
  Std_Error = M2_standard_errors
)


# Print the tables
print(table_model1)
print(table_model2)
```
