# Supplementary material for: Signalling strategies and opportunistic behaviour: Insights from dark-net markets
Source: PLoS One. 2025 Mar 18;20(3):e0319794. doi: 10.1371/journal.pone.0319794 (PMC11918442; doi:10.1371/journal.pone.0319794)
Supplement: S4 File — (PDF) [file pone.0319794.s007.pdf]

```
library(mgcv)
library(readr)
setwd("")
data <- read_csv("S1 data.csv")

M1 <- gam(percentage_of_frauds ~
               s(perc_positive_reviews) +  # Smooth function of percentage of positive
reviews
            s(n_of_reviews) +  # Smooth function of number of reviews
            price +  # Linear term for price
            s(days_selling) +  # Smooth function of days selling
            factor(shipment)+  # Include international shipment as predictor
            factor(product_category) +  # Include product category as predictor
            factor(product_class) +  # Include product class as predictor
            factor(method_of_payment),
          data = data,  # Use 'data' dataframe
          method = "REML",  # Use Restricted Maximum Likelihood for estimation
          family = tw())  # Use Tweedie distribution

summary(M1)

saveRDS(M1, file = "gam_model1.rds")
model1 <- readRDS("gam_model1.rds")

M2 <- gam(percentage_of_frauds ~
               s(perc_positive_reviews) +  # Smooth function of percentage of positive
reviews
            s(n_of_reviews) +  # Smooth function of number of reviews
            price +  # Linear term for price
            s(days_selling) +  # Smooth function of days selling
            factor(shipment)+  # Include international shipment as predictor
            factor(product_category) +  # Include product category as predictor
            factor(product_class) +  # Include product class as predictor
            factor(method_of_payment)+
            length_of_text+
            normalized_score+
            linguistic_diversity,
          data = data,  # Use 'data' dataframe
          method = "REML",  # Use Restricted Maximum Likelihood for estimation
          family = tw())  # Use Tweedie distribution

summary(M2)

saveRDS(M2, file = "gam_model2.rds")
model2 <- readRDS("gam_model2.rds")
```
